# Seasonal ovulatory activity exists in tropical Creole female goats and Black Belly ewes subjected to a temperate photoperiod

**DOI:** 10.1186/1472-6793-4-12

**Published:** 2004-08-27

**Authors:** Philippe Chemineau, Agnès Daveau, Yves Cognié, Gilles Aumont, Didier Chesneau

**Affiliations:** 1 Equipe de Neuroendocrinologie et Maîtrise des Fonctions Saisonnières, Unité de Physiologie de la Reproduction et des Comportements, UMR INRA-CNRS-Haras Nationaux-Univ. F. Rabelais, 37380 Nouzilly, France; 2 C.R.A.A.G. Station de Recherches Zootechniques, BP 1232, 94195 Pointe à Pitre cedex, France; 3 Departement Santé Animale 37380 Nouzilly, France

## Abstract

**Background:**

Seasonality of ovulatory activity is observed in European sheep and goat breeds, whereas tropical breeds show almost continuous ovulatory activity. It is not known if these tropical breeds are sensitive or not to temperate photoperiod. This study was therefore designed to determine whether tropical Creole goats and Black-Belly ewes are sensitive to temperate photoperiod. Two groups of adult females in each species, either progeny or directly born from imported embryos, were used and maintained in light-proof rooms under simulated temperate (8 to 16 h of light per day) or tropical (11 – 13 h) photoperiods. Ovulatory activity was determined by blood progesterone assays for more than two years. The experiment lasted 33 months in goats and 25 months in ewes.

**Results:**

Marked seasonality of ovulatory activity appeared in the temperate group of Creole female goats. The percentage of female goats experiencing at least one ovulation per month dramatically decreased from May to September for the three years (0%, 27% and 0%, respectively). Tropical female goats demonstrated much less seasonality, as the percentage of goats experiencing at least one ovulation per month never went below 56%. These differences were significant.

Both groups of temperate and tropical Black-Belly ewes experienced a marked seasonality in their ovulatory activity, with only a slightly significant difference between groups. The percentage of ewes experiencing at least one ovulation per month dropped dramatically in April and rose again in August (tropical ewes) or September (temperate ewes). The percentage of ewes experiencing at least one ovulation per month never went below 8% and 17% (for tropical and temperate ewes respectively) during the spring and summer months.

**Conclusions:**

An important seasonality in ovulatory activity of tropical Creole goats was observed when females were exposed to a simulated temperate photoperiod. An unexpected finding was that Black-Belly ewes and, to a lesser extent, Creole goats exposed to a simulated tropical photoperiod also showed seasonality in their ovulatory activity. Such results indicate that both species are capable of showing seasonality under the photoperiodic changes of the temperate zone even though they do not originate from these regions.

## Background

Seasonality of reproduction is a common feature in sheep and goat breeds of temperate latitudes [1,2] and seems to have been present for millennia in the sheep and goat breeding systems [3]. The annual breeding season begins in summer in Ile-de-France ewes and in autumn in Alpine goats and ends in winter in both species, resulting in a marked seasonality in birth dates of lambs and kids. In goats and sheep, this seasonality is under photoperiodic control. In experimental conditions, long days inhibit and short days stimulate sexual activity (goats: [4-6] sheep: [1]). However, under natural conditions of temperate countries, goats, as well as sheep [7,8], probably have an endogenous rhythm that is synchronized by photoperiod such that breeding occurs during autumn/winter and anovulation (anestrus) occurs during spring/summer.

When transferred to equatorial conditions (12 h of light per day, with a limited control of temperature change amplitudes), Suffolk ewes (a European breed) cycled at irregular intervals with no clear anovulatory season [9]. In contrast, when transferred to tropical photoperiodic conditions where the annual amplitude of photoperiodic changes exists but is lower than in temperate regimen, Alpine goats do not greatly modify the seasonal characteristics of their breeding season, and long periods of anestrus and anovulation are still present during spring and summer as in control females maintained under temperate photoperiod [10].

Local breeds of sheep [11-15] and goats [12,16,17] under their native tropical conditions, are either non-seasonal breeders or exhibit only a weak seasonality of reproduction. The females of these breeds ovulate and exhibit estrus almost the whole year round, even though short periods of anovulation and anestrus are detected in some females. Two main hypotheses can be raised to explain the near-absence of seasonality: either the females are insensitive to photoperiod, or the amplitude of the photoperiodic changes is too small. It is thus interesting to determine whether absence of seasonality persists when females of these breeds are subjected to major annual changes in the amplitude of day length, the prevailing conditions in temperate regions, or whether seasonality appears as it does in most temperate breeds.

In the present experiment, seasonal ovulatory activities were assessed in tropical Creole goats and Black-Belly ewes. These two breeds originate from the Carribean Islands, where they have been bred for 3–4 centuries, and constitute the progeny of African tropical breeds (see Methods). The animals of the present experiment were imported into Europe and experimentally subjected for more than two years in light-proof rooms to an annual photoperiodic regimen simulating that of temperate regions (TE group), and compared to females under tropical cycle simulating that of a tropical region (TR group).

## Results

### Ovulatory activity in Creole goats

**Figure 1:**
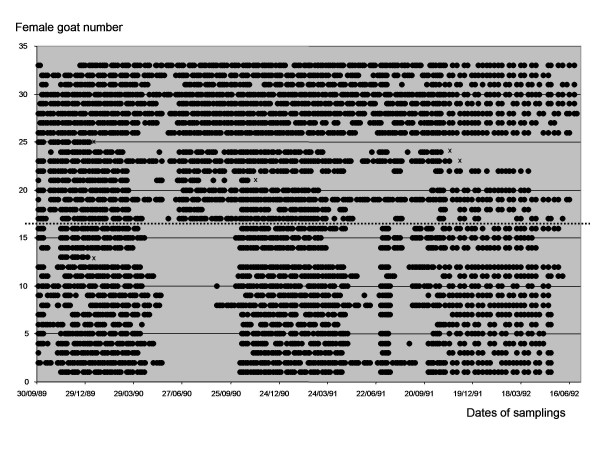
**Individual ovulatory activity in Creole goats maintained under two different photoperiodic conditions. **Two groups of Creole female goats were maintained in light-proof rooms and subjected to either simulated temperate photoperiodic conditions (TE goats 1-16 at the bottom of the figure, 8 to 16 hours of light per day from winter to summer solstice), or simulated tropical photoperiodic conditions (TR goats 17-33 upper part of the figure, 11 to 13 hours of light per day from winter to summer solstice) for 33 months. Ovulatory activity was assessed by twice-weekly (first 25 months) then once-weekly (last 8 months) determinations of plasma progesterone by radioimmunoassay. One line represents one female goat. A single dot represents one progesterone measurement above 1 ng/ml of plasma and is considered as indicative of the presence of at least one corpus luteum. Female goats which died or were excluded from the experiment are indicated by an "x".

Ovulatory activity demonstrated marked differences between experimental groups over the course of the experiment. Individual ovulatory activity is presented in Figure 1.

**Figure 2:**
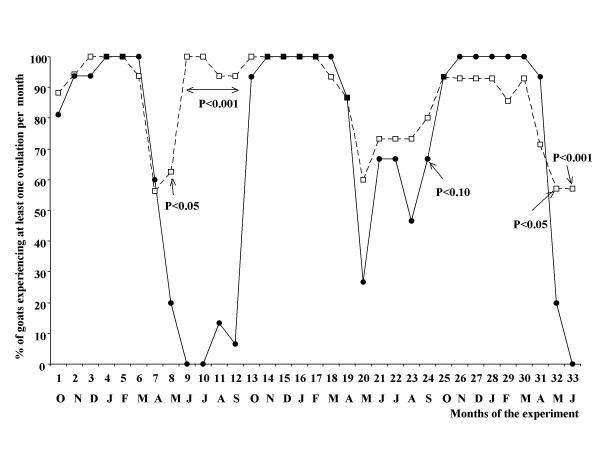
**Ovulatory activity in Creole goats maintained under two different photoperiodic conditions. **Two groups of Creole female goats were maintained in light-proof rooms and subjected to either simulated temperate photoperiodic conditions (TE solid line closed circles, 16 goats, 8 to 16 hours of light per day from winter to summer solstice), or simulated tropical photoperiodic conditions (TR dashed line open squares, 17 goats, 11 to 13 hours of light per day from winter to summer solstice) for 33 months. Ovulatory activity was assessed by twice-weekly (first 25 months), then once-weekly (last 8 months) determinations of plasma progesterone by radioimmunoassay. Results are expressed as the percentage of female goats experiencing at least one ovulation per month. Significant differences between groups are indicated.

Marked seasonality of ovulatory activity appeared in female goats of the TE group; the seasonal inactivity occurred from May to September for the three years of the study (Figures 1 &2). The percentage of female goats experiencing at least one ovulation per month dramatically decreased from May to September for the three years (0%, 27% and 0%, respectively). All female goats experienced an anovulatory period during the first spring/summer season (1990), only one of them continued its ovulatory activity in 1991, and all of them stopped again in May 1992.

In contrast, female goats exposed to the TR photoperiodic cycle demonstrated much less seasonality as the percentage of goats experiencing at least one ovulation per month never went below 56% (minima for the three years of study: 56%, 56%, 57%). Two female goats cycled continuously during the course of the experiment, one female goat in mid-1990 and three in mid-1991 continued their ovulatory activity during spring and summer, and four females were still cycling at the end of the experiment (June 1992).

These differences between the two groups led to significant differences in some, but not all, parameters. The percentage of goats experiencing at least one ovulation per month (Figure 2) was significantly lower in the TE than in the TR group in May (P < 0.05), June, July, August and September 1990 (P < 0.001), tended to be lower in May 1991 (P < 0.10), and was lower again in May (P < 0.05) and June 1992 (P < 0.001). In both groups the females which stopped their ovulatory activity did so at roughly the same date (April-May) in the 3 years of the experiment (Table 1). TR goats began their first breeding season significantly earlier than TE goats in 1990, but this difference did not appear in the second breeding season (Table 1). Variances of the dates of end of the 2^nd ^and 3^rd ^breeding seasons were significantly higher in TR goats (Table 1). The duration of the anovulatory period in 1990 was significantly shorter in TR than in TE goats and the duration of the 1990–1991 breeding season was significantly longer in TR than in TE goats (Table 2). The 1991 anovulatory period and the 1991–1992 breeding season did not differ between groups (Table 2). Variances of the duration of the 2^nd ^anovulatory season and of the 3^rd ^breeding season were significantly higher in TR goats (Table 2).

### Ovulatory activity in Black-Belly ewes

**Figure 3:**
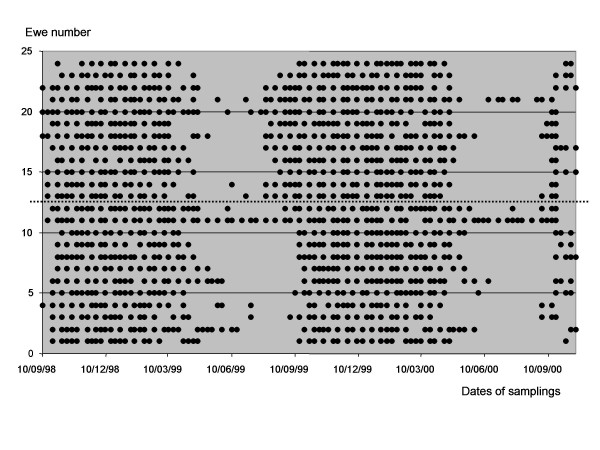
**Individual ovulatory activity in Black-Belly ewes maintained under two different photoperiodic conditions. **Two groups of Black-Belly ewes were maintained in light-proof rooms and subjected to either simulated temperate photoperiodic conditions (TE ewes 1-12 at the bottom of the figure, 8 to 16 hours of light per day from winter to summer solstice) or simulated tropical photoperiodic conditions (TR ewes 13-24 upper part of the figure, 11 to 13 hours of light per day from winter to summer solstice) for 25 months. Ovulatory activity was assessed by once-weekly determinations of plasma progesterone by radioimmunoassay. One line represents one ewe. A single dot represents one progesterone measurement above 0.75 ng/ml of plasma and is considered as indicative of the presence of at least one corpus luteum. All ewes were present until the end of the experiment.

Ovulatory activity demonstrated marked variations in both experimental groups over the course of the experiment. Individual ovulatory activity is presented in Figure 3.

**Figure 4:**
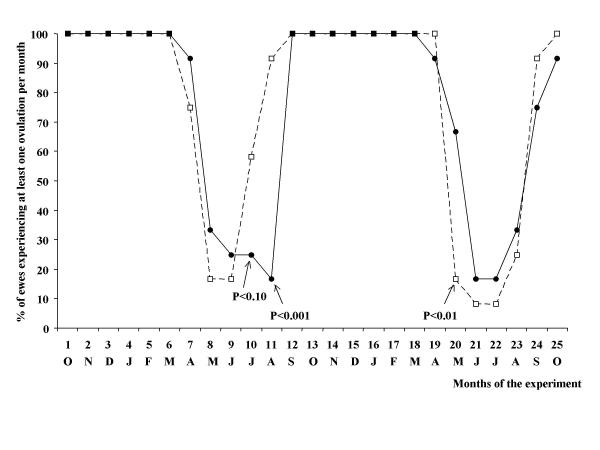
**Ovulatory activity in Black-Belly ewes maintained under two different photoperiodic conditions. **Two groups of Black-Belly ewes were maintained in light-proof rooms and subjected to either simulated temperate photoperiodic conditions (TE solid line closed circles, 12 ewes, 8 to 16 hours of light per day from winter to summer solstice) or simulated tropical photoperiodic conditions (TR dashed line open squares, 12 ewes, 11 to 13 hours of light per day from winter to summer solstice) for 25 months. Ovulatory activity was assessed by once-weekly determinations of plasma progesterone by radioimmunoassay. Results are expressed as the percentage of ewes experiencing at least one ovulation per month. Significant differences between groups are indicated.

From October to March of the first year, all females were cycling in both groups (100% of ewes showed at least one ovulation per month; Figure 3 &4). In the TR group, ovulatory activity dropped in April, remained low (2 ewes cycling) in May and June, then rose again in July and August to reach its maximum from September to April of the next year; minimum activity was observed again from May to August, before a maximal activity in September and October. Ewes of the TE group roughly followed the same pattern, with a later onset of cyclicity in September of the first year (% of ewes cycling in August P < 0.001); and a later end in May-June of the second year (% of ewes cycling in May P < 0.01). The percentage of cycling females never went below 17% (2 ewes cycling). One ewe in the TE group never stopped cycling during the course of the experiment.

Mean dates of the end of the breeding season did not differ between TR and TE ewe groups in the first and second year (April, Table 3). The onset of the breeding season occurred earlier in TR than in TE for the first year, but not for the second year (Table 3). Thus, duration of the first and second anestrous seasons and/or duration of the sexual season did not differ between TR and TE ewe groups (Table 4). Variances of the durations of anovulatory and of breeding seasons were significantly higher in TE ewes (Table 4).

## Discussion

The two tropical breeds of Creole goats and Black-Belly ewes used in the present study demonstrated clear seasonal breeding activity with a definite cut-off when maintained under the simulated extensive photoperiodic variations of temperate areas. As many other goat and sheep breeds originating and raised under a temperate climate, these two breeds imported from the tropics displayed cessation of ovulatory activity in spring and summer, i.e. the usual months for anovulation and anestrus in a temperate climate.

Even though it appears that their anovulatory season seemed shorter than European breeds of goats [e.g. Alpine, [10]] and sheep [e.g.. Ile-de-France, [18]], almost all Black-Belly ewes stopped their ovulatory activity for about 4 months and Creole goats for 2.5 months. Black-Belly ewes stopped their ovulatory activity late in the year (second half of April) as compared to the majority of breeds, for example Ile-de-France breed [mid-January; [18]) or the majority of British breeds [19]. On the other hand, they started their breeding season later that these breeds, showing a more "primitive" type of breeding season, similar to those displayed by the Moufflon [20], Romanov [21] or Icelandic [22] breeds of sheep. A similar observation could be made for the end of the breeding season of the Creole goats maintained under simulated temperate photoperiod: they stopped late in the season (April-May) compared to temperate breeds [February, [10]]. However, this was not true for the onset of the breeding season which generally started in September-October in both goat breeds.

The control group of goats maintained under simulated tropical photoperiodic variations displayed significantly less seasonality. The percentage of goats showing at least one ovulation per month was significantly higher in May and June for 2 years out of 3, and did not drop to 0 as it did in the temperate group of goats. A relatively high number of female goats did not experience an anovulatory season either at all or during some years of the experiment, and those that did showed a significantly shorter anovulatory period during the first year of the experiment.

Thus, the comparison between Creole female goats maintained under simulated temperate photoperiod and control females placed under simulated tropical photoperiod leads to the conclusion that their breeding season is sensitive to large photoperiodic variations.

In contrast, Black-belly ewes maintained under simulated tropical photoperiodic variations did not differ greatly from those maintained under simulated temperate photoperiod. The two groups of ewes and goats used here differed in various parameters, some of which could explain the photoperiod x species interaction observed here: (a) Recipient ewes in which embryos were implanted in autumn were maintained under natural photoperiod. It is known that light changes during pregnancy may affect the progeny's photoperiod sensitivity, especially regarding the onset of puberty in sheep [23-25] and in rodents [26]. This was not the case in the goats, as these experimental animals were the 3^nd ^or 4^th ^progeny of females imported as embryos. (b) Experimental ewes were artificially raised in TE *vs *TR photoperiod from birth to 6 months old from the start of the experiment, whereas female goats were raised under simulated tropical photoperiod until the start of the experiment at one or two years old. (c) Black-Belly ewes were included in the experiment at 6 months of age, whereas Creole goats were 12 and 24 months old at the start of the experiment. These three main differences between the experiments in female goats and ewes could possibly explain this photoperiod x species interaction. However, it may also come from a real difference of sensitivity to non-photoperiodic factors (such as temperature changes or activity, see later) between the two species.

The spontaneous ovulatory activity demonstrated in the present experiment by the Black-Belly ewes from the TR group were very different from those registered earlier in their natural breeding conditions on the island of Martinique in the tropics, where they cycled all year round [15]. The Creole goats from the simulated tropical photoperiod did not display here the same results as females of the same flock raised in their natural breeding conditions on the island of Guadeloupe in the tropics, where they also cycled almost continuously [17]. This unexpected difference suggests that other external cues may act in combination with photoperiod to inhibit breeding activity. The fact that all animals simultaneously stopped cycling in spring, suggests that an external physical cue could be involved. The temperature of the light-proof rooms in which the experiments were performed was not controlled and the high and low-amplitude variations of air temperature of the tropics were not applied to our experimental animals. Such a cue may interact with photoperiod and enhance the negative effects of the limited photoperiodic changes, which do not appear in normal tropical conditions. To our knowledge, very few experiments have been carried out in sheep and/or goats to determine the role played by low temperature in the appearance of seasonality. It has been demonstrated that inversion of the temperature rhythm does not entrain ovulatory activity in ewes of a European breed maintained under an equatorial photoperiodic schedule [27] and that low temperatures in the summer time may induce an 8-week advance in the onset of the annual breeding season in dark-faced ewes [28]. In Suffolk ewes, a seasonal breed, the maintenance of ewes under an equatorial regimen with a limited but efficient control of temperature change amplitude, induced cycles at irregular intervals with no clear anovulatory season [9] However, in other species it has been clearly demonstrated that the combination of photoperiod and temperature is responsible for seasonal changes in reproductive activity [29,30]. Thus, it is possible that, in the absence of a major cue (photoperiod), seasonal ovulatory activity of females of the TR groups has been entrained by temperature.

In Syrian hamsters, exercise by access to a running wheel can completely inhibit the short-day induced regression of the testis [31,32]. Experimental Creole goats and Black Belly ewes of the present experiment were raised in light-proof rooms where physical exercise was limited, whereas in their original management conditions where the initial observations were done [15,17], animals were maintained at pasture. Thus, it is possible that in their original management conditions, physical exercise prevented the inhibitory effects of the 13 hours of light that was observed in our experimental light-proof rooms in the TR groups of goats and ewes.

The sheep and goat breeds used here are local breeds of the Carribean islands. Even though their presence in these islands is associated with human history, they are considered as the progeny of tropical but not European ancestors, because they originate from the West coast of tropical Africa (see Methods). In these areas all sheep and goat breeds are considered as non or low seasonal breeders. Thus, the sensitivity of the Creole goats and Black-Belly ewes observed here under temperate photoperiod could be hypothetized as a true sensitivity of these breeds, not a simple inheritance of a trait coming from European ancestors.

## Conclusions

A marked seasonality in the ovulatory activity of tropical Creole goats and Black-Belly ewes was induced when females were exposed to a simulated temperate photoperiod. Unexpectedly, and differing from the results obtained in their original breeding location, Black-Belly ewes and, to a lesser extent, Creole goats exposed to a simulated tropical photoperiod also showed significant seasonality in their ovulatory activity. Such results indicate that both species are capable of showing seasonality under the the photoperiodic changes of the temperate zone even though they do not originate from these regions.

## Methods

### Production of experimental animals from deep-frozen embryos

Experimental animals from both species were produced after importation of deep-frozen embryos. The embryos were thawed and re-implanted into recipient females of Saanen goats for Creole embryos (1983) and of Ile-de-France sheep for Black-Belly embryos (1997).

Embryos from donor Creole goats were collected as previously described [33] from 4 genetic families considered as representative of the native population of the Caribbean island of Guadeloupe (French West Indies). This breed has been raised in Guadeloupe for several centuries and probably originates from the West coast of Africa which it was imported from in the 17^th^-18^th ^centuries [34-37]. Creole goats from Guadeloupe have many similarities with the "West African dwarf goat" regarding their size, coat color, fertility and prolificacy, growth rate and horn shapes [34,37]. Common genetic markers were found between Creole goat from Guadeloupe and West African goats [36], which reinforced the hypothesis of an African origin for the Creole goat. The first generation of animals, originating from deep-frozen imported embryos, was not used in the experiment. They were raised, with their progeny, under tropical photoperiodic conditions in light-proof buildings, as described later. The 3^rd ^and 4^th ^generations, constituting a sufficient number of animals, made up the two experimental groups.

Embryos from donor Black-Belly ewes were collected using the technique described by Heyman et al. [38]. The donor females belonged to an INRA flock raised in Guadeloupe and were part of the 6 different families constituting this flock, bred from genitors from Martinique (F.W.I.) [39,40] and Barbados. This flock was considered to present characteristic production traits of the Black-Belly sheep population of the Caribbean [41]. As for goats, Black-Belly sheep is considered to have an African origin about 3 to 4 centuries ago [11,42]. This is confirmed by their phenotypic characteristics of hair sheep (i.e. not wool sheep), including performance traits [43]. After checking the absence of Blue-tongue virus in the collection media, embryos were re-implanted into Ile-de-France ewe lambs, 2 embryos inserted per recipient ewe. In the case of sheep, the first generation originating from deep-frozen embryos was used directly in the present experiment. After birth, all lambs were immediately placed under artificial suckling conditions, in the two experimental groups in light-proof rooms under tropical or temperate photoperiod, until the start of blood sampling for progesterone determinations.

### Animals and feeding conditions

Both experiments were performed at the INRA Station near Tours (latitude 47°25 North).

- Thirty three Creole female goats were used from October 1989 when the animals were one (n = 15 females) and two (n = 18 females) years old, for 33 months to June 1992 . They were divided into two groups (n = 17 TR and 16 TE) in visual and tactile contact, with entire and vasectomized Creole bucks, but separated by a fence. Each group was maintained in a separate light-proof room throughout the experiment.

Feeding conditions were constant throughout the experiment. The animals were fed once daily with a diet of 240 g of barley, 60 g of wheat, 700 g of meadow hay and 300 g of barley straw. No flushing was performed. They had free access to water and to mineral blocks containing oligoelements and vitamins.

- Twenty four Black Belly ewes were used from September 1998 when the animals were 6 months old, for 25 months, to October 2000. They were divided into two groups (n= 12 TR and 12 TE) in visual and tactile contact, with entire Black Belly rams, but separated by a fence. Each group was maintained in a separate light-proof room throughout the experiment.

Feeding conditions were constant throughout the experiment. The animals were fed once daily with a diet of 150 g of corn, 110 g of barley, 45 g of dehydrated protein complement and 400 g of hay. No flushing was performed. They had free access to water and to mineral blocks containing oligoelements and vitamins.

### Photoperiodic treatments

Within each species, one group was subjected to the large photoperiodic changes prevailing at the 45° North latitude (8 to 16 h of light per day from winter to summer solstices); this group was called the temperate group (TE). The other group was subjected to the limited photoperiodic changes prevailing at the 16° North latitude (11 to 13 h of light per day from winter to summer solstices); this group was called the tropical group (TR). In all rooms, photoperiod was regulated by an electric clock that operated bulbs providing an intensity of 300 lux, lateral to the animals' eyes. The photoperiod was adjusted by 15 min shifts (more or less frequent depending on the slope of the natural changes in daylength) to produce a complete photoperiodic cycle every 365 days.

The four rooms were adjacent and of the same size (30 m^2^). Temperature was not controlled but variations were parallel to those monitored outside but with a lower amplitude (minimum +1°C in January, maximum +29°C in August).

### Measurements and samplings

Liveweight of Creole goats at the beginning of the experiment was 24.6 (± 3.4, sd) kg in group TE and 25.5 (± 3.0) kg in group TR. Liveweight was measured monthly and showed a regular increase until the end of the experiment (55.7 ± 7.5 and 56.2 ± 5.2 kg for TE and TR respectively). Liveweight of Black-Belly at the beginning of the experiment was 33.2 (± 2.5 sd) kg in group TE and 31.9 (± 2.7) kg in group TR. Liveweight was measured monthly and showed a regular increase until the end of the experiment (49.5 ± 5.9 and 47.0 ± 5.8 kg for TE and TR respectively).

Ovulatory activity was assessed twice weekly in goats up to end October 1991 and once weekly thereafter; and once weekly in ewes, using blood samples for the progesterone radioimmunoassay. A rapid assay was performed using the technique described by Terqui and Thimonier [44]. When progesterone concentration was lower than 1.0 ng per ml of plasma in female goats and 0.75 ng/ ml of plasma in ewes, the female was considered to be in the follicular phase of the cycle or in anovulation.

### Definitions and analysis of results, statistical tests

The first occurrence of a positive Progesterone sample was considered as the date of the first ovulation of the season, and the last occurrence of a positive Progesterone sample was considered as the date of the last ovulation of the season. Mean duration of ovulatory activity is the number of days between first and last ovulation in the same breeding season. Mean duration of anovulation is the number of days between last ovulation in a breeding season and first ovulation of the next season. Females which did not present cessations of their ovulatory activity were not included in the calculations of duration of the breeding seasons or duration of the anovulatory periods. In September and October 2000, individual blood samplings were stopped in ewes that had resumed their ovulatory activity.

Mean dates of onset and end of the breeding season, durations of the breeding season and of anovulatory activity were compared between groups using an unpaired t-test. Variances were compared with F-Tests. Percentages of females showing at least one ovulation per month were analyzed using the Chi^2 ^method. (Statview^®^, Abacus Concept, Berkeley, Ca, USA).

All procedures were performed in accordance with French legal requirements and with the Ministry of Agriculture authorization for animal experimentation nb A37801 .

## Authors' contributions

The authors contributed equally to this work. PC conceived the study, and was responsible for its design and coordination. AD followed the experiment in goats and DC the experiment in sheep. YC and GA performed all the embryo transfer procedures, and were in charge of pathological analyses. AD, DC and PC analysed the data. PC drafted the manuscript. All authors read, corrected and approved the final manuscript.

## Figures and Tables

**Table 1:** Mean dates ± S.E.M. in days, for onset and end of ovulatory activity in Creole goats maintained under two different photoperiodic conditions.

	1^st ^breeding season 1989–1990		2^nd ^breeding season 1990–1991		3^rd ^breeding season 1991–1992	
	onset	end	onset	end	onset	end

Temperate regimen	*NA*	20/04 ± 4	10/10 ± 11	22/04 ± 5	11/07 ± 7	08/05 ± 4
Tropical regimen	*NA*	14/04 ± 7	18/6*** ± 8**	02/05 ± 11*	23/07 ± 15**	02/05 ± 12**

Two groups of Creole female goats were maintained in light-proof rooms and subjected to either simulated temperate photoperiodic conditions (TE, 16 goats, 8 to 16 hours of light per day from winter to summer solstice), or simulated tropical photoperiodic conditions (TR, 17 goats, 11 to 13 hours of light per day from winter to summer solstice) for 33 months. Ovulatory activity was assessed by determinations of plasma progesterone by radioimmunoassay. Means and variances were compared between groups within each column. Significant differences between means and between variances are indicated by asterisks (*:p < 0.05, **: P < 0.01, ***: P < 0.001). NA: not available.

**Table 2:** Mean durations in days ± S.E.M. of anovulatory and ovulatory seasons in Creole goats maintained under two different photoperiodic conditions.

	1^st ^anovulatory season (1990)	2^nd ^anovulatory season (1991)	2^nd ^breeding season (90–91)	3^rd ^breeding season (91–92)
Temperate regimen	173.0 ± 8.0	79.6 ± 9.5	194.5 ± 9.5	301.8 ± 7.8
Tropical regimen	65.0*** ± 8.4	81.6 ± 21.6*	304.3*** ± 10.8	265.5 ± 31.3***

Two groups of Creole female goats were maintained in light-proof rooms and subjected to either simulated temperate photoperiodic conditions (TE, 16 goats, 8 to 16 hours of light per day from winter to summer solstice), or simulated tropical photoperiodic conditions (TR, 17 goats, 11 to 13 hours of light per day from winter to summer solstice) for 33 months. Ovulatory activity was assessed by determinations of plasma progesterone by radioimmunoassay. Means and variances were compared between groups within each column. Significant differences between means and between variances are indicated by asterisks (*:p < 0.05, ***: P < 0.001).

**Table 3:** Mean dates ± S.E.M. in days, for onset and end of ovulatory activity in Black-Belly ewes maintained under two different photoperiodic conditions.

	1^st ^breeding season 1998–1999		2^nd ^breeding season 1999–2000		3^rd ^breeding season 2000–2001	
	onset	end	onset	end	onset	end

Temperate regimen	*NA*	22/04 ± 7	15/09 ± 3	25/04 ± 12	22/09 ± 5	*NA*
Tropical regimen	*NA*	09/04 ± 4	05/08*** ± 5*	22/04 ± 4*	13/09 ± 3	*NA*

Two groups of Black-Belly ewes were maintained in light-proof rooms and subjected to either simulated temperate photoperiodic conditions (TE, 12 ewes, 8 to 16 hours of light per day from winter to summer solstice) or simulated tropical photoperiodic conditions (TR, 12 ewes, 11 to 13 hours of light per day from winter to summer solstice) for 25 months. Ovulatory activity was assessed by determinations of plasma progesterone by radioimmunoassay. Means and variances were compared between groups within each column. Significant differences between means and between variances are indicated by asterisks (*:p < 0.05, ***: P < 0.001). NA: not available.

**Table 4:** Mean durations in days ± S.E.M. of anovulatory and ovulatory seasons in Black-Belly ewes maintained under two different photoperiodic conditions.

	1^st ^anovulatory season (1999)	2^nd ^anovulatory season (2000)	2^nd ^breeding season (99–2000)

Temperate regimen	134.2 ± 14.0	134.3 ± 14.0	237.8 ± 13.3
Tropical regimen	118.4 ± 7.5*	144.3 ± 5.8*	260.6 ± 6.7*

Two groups of Black-Belly ewes were maintained in light-proof rooms and subjected to either simulated temperate photoperiodic conditions (TE, 12 ewes, 8 to 16 hours of light per day from winter to summer solstice) or simulated tropical photoperiodic conditions (TR, 12 ewes, 11 to 13 hours of light per day from winter to summer solstice) for 25 months. Ovulatory activity was assessed by determinations of plasma progesterone by radioimmunoassay. Means and variances were compared between groups within each column. Significant differences between means and between variances are indicated by asterisks (*:p < 0.05).

## References

[B1] Ortavant R, Pelletier J, Ravault JP, Thimonier J, Volland P (1985). Photoperiod: main proximal and distal factor of the circannual cycle of reproduction in farm mammals. Oxf Reprod Rev Biol.

[B2] Shelton M (1978). Reproduction and breeding of goats. J Dairy Sci.

[B3] Balasse M, Smith AB, Ambrose SH, Leigh SR (2003). Determining sheep birth seasonality by analysis of tooth enamel oxygen isotope ratios: The Late Stone Age site of Kasteelberg (South Africa). J Archaeol Sci.

[B4] Bissonnette TH (1941). Experimental modification of breeding cycle in goats. Physiol Zool.

[B5] Chemineau P, Martin GB, Saumande J, Normant E (1988). Seasonal and hormonal control of pulsatile LH secretion in the dairy goat (*Capra hircus*). J Reprod Fertil.

[B6] Mori Y, Tanaka M, Maeda K, Hoshino K, Kano Y (1987). Photoperiodic modification of negative and positive feedback effects of oestradiol on LH secretion in ovariectomized goats. J Reprod Fertil.

[B7] Karsch FJ, Bittman EL, Foster DL, Goodman RL, Legan SJ, Robinson JE (1984). Neuroendocrine basis of seasonal reproduction. Recent Prog Horm Res.

[B8] Malpaux B, Migaud M, Tricoire H, Chemineau P (2001). Biology of mammalian photoperiodism and the critical role of the pineal gland and melatonin. J Biol Rhythms.

[B9] Jansen HT, Jackson GL (1993). Circannual rhythms in the ewe: patterns of ovarian cycles and prolactin secretion under two different constant photoperiods. Biol Reprod.

[B10] Chemineau P, Daveau A, Maurice F, Delgadillo JA (1992). Seasonality of oestrus and ovulation is not deeply modified by submitting Alpine goats to a tropical photoperiod. Small Rumin Res.

[B11] Mason JL (1980). Les ovins tropicaux prolifiques. Etude FAO: Production et Santé Animales.

[B12] Charray J, Coulomb J, Haumesser JB, Planchenault D, Pugliese PL, Provost A, IEMVT-Minist Coop Paris (1980). Les petits ruminants d'Afrique Centrale et d'Afrique de l'Ouest.

[B13] Yenikoye A (1984). Annual variations in estrual behavior, rate and possibilities for ovulation in Peulh ewes from Niger. Reprod Nutr Dev.

[B14] Gonzalez-Stagnaro C, Chemineau P, Gauthier D, Thimonier J (1983). Comportamiento reproductivo de las razas locales de rumiantes en el tropico americano. In Reproduction des ruminants en zone tropicale.

[B15] Mahieu M, Jego Y, Driancourt MA, Chemineau P (1989). Reproductive performances of Creole and Black-Belly ewes in the West Indies. A new major gene controlling ovulation rate?. Anim Reprod Sci.

[B16] Sutherland SRD (1988). Seasonal breeding and oestrus in the female goats. PhD Thesis Univ Western Australia.

[B17] Chemineau P (1986). Sexual behaviour and gonadal activity during the year in the tropical Creole meat goat. I. Female oestrous behaviour and ovarian activity. Reprod Nutr Dev.

[B18] Thimonier J, Mauléon P (1969). Variations saisonnières du comportement d'oestrus et des activités ovariennes et hypophysaires chez les ovins. Biol Anim Biochim Biophys.

[B19] Hafez ESE (1952). Studies on the breeding season and reproduction of the ewe. Agric Sci.

[B20] Santiago-Moreno J, Lopez-Sebastian A, Gonzalez-Bulnes A, Gomez-Brunet A, Tortonese D (2001). The timing of the onset of puberty, extension of the breeding season, and length of postpartum anestrus in the female mouflon (Ovis gmelini musimon). J Zoo Wildl Med.

[B21] Thimonier J (1989). Contrôle photopériodique de l'activité ovulatoire chez la brebis. Existence de rythmes endogènes. PhD Thesis Univ F Rabelais Tours Fr.

[B22] Dyrmundsson OR (1978). Studies on the breeding season of Icelandic ewes and ewe lambs. J Agric Sci.

[B23] Herbosa CG, Wood RI, I'Anson H, Foster DL (1994). Prenatal photoperiod and the timing of puberty in the female lamb. Biol Reprod.

[B24] Sunderland SJ, O'Callaghan D, Boland MP, Roche JF (1995). Effect of photoperiod before and after birth on puberty in ewe lambs. Biol Reprod.

[B25] Helliwell RJ, Wallace JM, Aitken RP, Racey PA, Robinson JJ (1997). The effect of prenatal photoperiodic history on the postnatal endocrine status of female lambs. Anim Reprod Sci.

[B26] Goldman BD (2003). Pattern of melatonin secretion mediates transfer of photoperiod information from mother to fetus in mammals. Sci STKE.

[B27] Wodzicka-Tomaszweska M, Hutchinson JCD, Bennett JW (1967). Control of the annual rhythm of breeding in ewes: effect of an equatorial daylength with reversed thermal seasons. J Agric Sci.

[B28] Dutt RH (1960). Temperature and light as factors in reproduction among farm animals. J Dairy Sci.

[B29] Larkin JE, Freeman DA, Zucker I (2001). Low ambient temperature accelerates short-day responses in Siberian hamsters by altering responsiveness to melatonin. J Biol Rhythms.

[B30] Larkin JE, Jones J, Zucker I (2002). Temperature dependence of gonadal regression in Syrian hamsters exposed to short day lengths. Am J Physiol Regul Integr Comp Physiol.

[B31] Gibbs FP, Petterborg LJ (1986). Exercise reduces gonadal atrophy caused by short photoperiod or blinding of hamsters. Physiol Behav.

[B32] Menet JS, Vuillez P, Saboureau M, Pévet P Inhibition of hibernation by exercise is not affected by intergeniculate leaflets lesion in hamsters. Physiol Regul Integr Comp Physiol.

[B33] Chemineau P, Procureur R, Cognie Y, Lefevre PC, Locatelli A, Chupin D (1986). Production, freezing and transfer of embryos from a bluetongue-infected goat herd without bluetongue transmission. Theriogenology.

[B34] Labat P, Guillaume Cavellier père, Paris (1792). Nouveau voyage aux Isles d'Amérique.

[B35] Chemineau P, Cognie Y, Xande A, Peroux F, Alexandre G, Levy F, Shitalou E, Beche JM, Sergent D, Camus E, Barre N, Thimonier J (1984). Le "cabrit créole" de Guadeloupe et ses caractéristiques zootechniques : monographie. Elev Méd Vét Pays Trop.

[B36] Pépin L (1994). Recherche de polymorphisme génétique chez les caprins. Application à l'étude de la diversité des populations, au contrôle de filiation et à la résistance génétique à la cowdriose. Thèse Univ Paris Sud, Orsay.

[B37] Alexandre G, Aumont G, Mandonnet N, Fleury J, Naves M (1999). La chèvre Créole de Guadeloupe (F. W. I.) :une ressource génétique importante pour les Tropiques humides. Bulletin d'Information sur les Ressources Génétiques Animales Paris.

[B38] Heyman Y, Vincent C, Garnier V, Cognié Y (1987). Transfer of frozen-thawed embryos in sheep. Vet Rec.

[B39] Bastien O, Matheron G, Leimbacher F (1991). Le mouton en Martinique. I. Description des principaux phénotypes identifiés et étude de quelques caractères morphologiques. Rev Elev Méd Vét Pays Trop (Supp).

[B40] Mahieu M, Aumont G, Alexandre G (1997). Élevage intensif des ovins tropicaux à la Martinique. INRA Prod Anim.

[B41] Thomas DL, Wildeus S (1991). Hair sheep genetic resources of the Americas (review paper). In Proceedings Hair Sheep Research Symposium, Ste Croix USVI.

[B42] Fitzhugh HA, Bradford GE (1983). Hair Sheep of Western Africa and the Americas. Westview Press, Boulder, Co, USA.

[B43] Naves M, Alexandre G, Leimbacher F, Mandonnet N, Menendez Buxadera A (2001). Le point sur les programmes de gestion des ressources génétiques chez les espèces de ruminants dans la Caraïbe. INRA Prod Anim.

[B44] Terqui M, Thimonier J (1974). Nouvelle méthode radioimmunologique rapide pour l'estimation du niveau de progestérone plasmatique. Application pour le diagnostic précoce de gestation chez la brebis et la chêvre. C R Acad Sci D Sci Vie.

